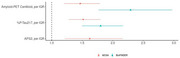# Association of Plasma Biomarkers of Alzheimer's Disease with Incident Tau PET Positivity

**DOI:** 10.1002/alz70856_098904

**Published:** 2025-12-24

**Authors:** Jonathan Graff‐Radford, Jeremy A. Syrjanen, Prashanthi Vemuri, Val J Lowe, Heather J. Wiste, Christopher G Schwarz, Alicia Algeciras‐Schimnich, Terry M. Therneau, Alexa Pichet Binette, Ruben Smith, Niklas Mattsson‐Carlgren, Sebastian Palmqvist, Erik Stomrud, David S. Knopman, Petrice M Cogswell, Ronald Petersen, Oskar Hansson, Clifford R. Jack

**Affiliations:** ^1^ Department of Neurology, Mayo Clinic, Rochester, MN, USA; ^2^ Mayo Clinic, Rochester, MN, USA; ^3^ Department of Radiology, Mayo Clinic, Rochester, MN, USA; ^4^ Department of Health Sciences Research, Mayo Clinic, Rochester, MN, USA; ^5^ Clinical Memory Research Unit, Department of Clinical Sciences, Lund University, Lund, Scania, Sweden; ^6^ Clinical Memory Research Unit, Lund University, Malmö, Skåne, Sweden; ^7^ Clinical Memory Research Unit, Department of Clinical Sciences Malmö, Faculty of Medicine, Lund University, Lund, Sweden; ^8^ Memory Clinic, Skåne University Hospital, Malmö, Skåne, Sweden; ^9^ Clinical Memory Research Unit, Lund University, Lund, Sweden; ^10^ Clinical Memory Research Unit, Department of Clinical Sciences Malmö, Faculty of Medicine, Lund University, Sweden, Lund, Sweden

## Abstract

**Background:**

The objective of this study was to determine the predictive value of amyloid‐PET versus plasma %p‐tau217 (defined as the ratio of phosphorylated to non‐phosphorylated Tau217 × 100), Aβ42/40, and the amyloid probability score (APS2; which combines of Aβ42/40 and %p‐tau217) in tau‐PET transitions (T‐ to T+).

**Method:**

Participants included were from the Mayo Clinic Study of Aging (MCSA) and had plasma markers measured by mass spectrometry (IP‐MS; C2N), had an amyloid‐PET scan, and had a tau‐PET negative scan (SUVR<1.29, temporal meta‐ROI) at the baseline, along with one or more follow‐up tau‐PET scans. Cox proportional hazard models were used to evaluate the relationship between C2N plasma %P‐Tau217 and APS2 and incident tau‐PET positivity (SUVR>=1.29) adjusting for age, sex, and *APOE* ε4. An analysis was also performed using amyloid‐PET centiloid as the predictor. Both the plasma markers and amyloid‐PET centiloid were scaled so that a one‐unit change corresponds to the respective interquartile range (IQR) for comparability of hazard ratios (HR). The analyses were repeated in the BioFINDER‐2 cohort for validation.

**Result:**

255 MCSA participants (122 female [47.8%] were included with a median age of 71.9 (IQR: 65.8, 78.8) years old, of which 37 progressed to tau‐PET positivity. At baseline, 238 (93.7%) were cognitively unimpaired. Higher baseline %p‐tau217 (HR: 1.52 [95% CI: 1.28, 1.8]), amyloid‐PET centiloid (HR: 1.47 [95% CI: 1.20, 1.79]), and APS2 (HR: 1.62 [95% CI: 1.22, 2.16]) were associated with an increased risk of progressing to tau‐PET positive (Figure 1). Aβ42/40 (HR: 1.06 [95% 0.604–1.867] was not associated with incident tau positivity Among 605 participants who were tau negative at index date in BioFINDER‐2 (316 (52.2%) cognitively unimpaired), with a median age of 70.2 (IQR: 60.5‐76.4), 33 progressed to tau positivity with higher %p‐tau217 (HR: 1.8 [95% CI: 1.5, 2.17]), amyloid‐PET centiloid (HR: 2.3 [95% CI: 1.77, 2.97]), and lower Aβ 42/40 (HR: 0.42 [95% CI: 0.21, 0.85]) at index date all associated with an increased risk of progressing to tau‐PET positive.

**Conclusion:**

In two independent cohorts**,** %p‐tau217 provides predictive information about those who will convert from tau negative to tau positive on PET scan with the associations being similar to amyloid‐PET.